# A20 Restricts Inflammatory Response and Desensitizes Gingival Keratinocytes to Apoptosis

**DOI:** 10.3389/fimmu.2020.00365

**Published:** 2020-03-10

**Authors:** Yajie Li, Erin C. Mooney, Xia-Juan Xia, Nitika Gupta, Sinem Esra Sahingur

**Affiliations:** ^1^Department of Periodontics, School of Dental Medicine, University of Pennsylvania, Philadelphia, PA, United States; ^2^School of Dentistry, Philips Institute for Oral Health Research, Virginia Commonwealth University, Richmond, VA, United States

**Keywords:** A20, NF-κB, epithelial cells, *Porphyromonas gingivalis*, cytokines, apoptosis, periodontitis

## Abstract

The pathophysiology of periodontal disease involves a perturbed immune system to a dysbiotic microflora leading to unrestrained inflammation, collateral tissue damage, and various systemic complications. Gingival epithelial cells function as an important part of immunity to restrict microbial invasion and orchestrate the subsequent innate responses. A20 (TNFAIP3), an ubiquitin-editing enzyme, is one of the key regulators of inflammation and cell death in numerous tissues including gastrointestinal tract, skin, and lungs. Emerging evidence indicates A20 as an essential molecule in the oral mucosa as well. In this study, we characterized the role of A20 in human telomerase immortalized gingival keratinocytes (TIGKs) through loss and gain of function assays in preclinical models of periodontitis. Depletion of A20 through gene editing in TIGKs significantly increased IL-6 and IL-8 secretion in response to *Porphyromonas gingivalis* infection while A20 over-expression dampened the cytokine production compared to A20 competent cells through modulating NF-κB signaling pathway. In the subsequent experiments which assessed apoptosis, A20 depleted TIGKs displayed increased levels of cleaved caspase 3 and DNA fragmentation following *P. gingivalis* infection and TNF/CHX challenge compared to A20 competent cells. Consistently, there was reduced apoptosis in the cells overexpressing A20 compared to the control cells expressing GFP further substantiating the role of A20 in regulating gingival epithelial cell fate in response to exogenous insult. Collectively, our findings reveal first systematic evidence and demonstrate that A20 acts as a regulator of inflammatory response in gingival keratinocytes through its effect on NF-κB signaling and desensitizes cells to bacteria and cytokine induced apoptosis in the oral mucosa. As altered A20 levels can have profound effect on different cellular responses, future studies will determine whether A20-targeted therapies can be exploited to restrain periodontal inflammation and maintain oral mucosa tissue homeostasis.

## Introduction

Periodontitis is the consequence of a deregulated immune response initiated by a dysbiotic oral microflora compromising the integrity of the periodontium and associated with a variety of systemic conditions (1–3). Locally, this uncontrolled inflammatory response results in the loss of epithelial attachment and connective tissue, as well as alveolar bone. While being recognized as one of the most common chronic inflammatory diseases affecting almost half of the adult population, severe and persistent forms of periodontitis are still able to resist conventional therapeutics and periodontitis continues to be a substantial medical, psychological, and financial burden worldwide affecting more than 50% of adult population. The recognition and response to oral microbiome is mainly driven by Toll-like receptors (e.g., TLR-2, TLR-4, and TLR-9) and the cooperative activity of the oral microbial community and host response is the major driver of oral mucosal tissue homeostasis (3). Numerous signaling cascades downstream of the TLRs, including nuclear factor kappa-light-chain-enhancer of activated B cells (NF-κB), trigger the release of inflammatory molecules critical for the activation of innate immunity, and subsequent development of the adaptive immune response. Although the immune response requires these multi-effect cytokine and chemokine signals to confine infections, prolonged exposure can lead to excessive inflammatory cell migration, secretion of tissue-damaging metalloproteases, and interference with key biological processes such as cell proliferation and apoptosis (2, 3). It is therefore important to understand the downstream signaling pathways and molecules that regulate timely termination of inflammation to prevent the damaging effect of unrestrained immune responses and maintain tissue homeostasis.

A20 (also known as tumor necrosis factor-α-induced protein 3 or TNFAIP3) is a pleiotropically expressed ubiquitin editing enzyme capable of interfering with NF-κB, interferon regulatory factor (IRF) and c-jun N-terminal (JNK) signaling cascades, all of which are being implicated with periodontal disease pathogenesis (4, 5). A20 serves as a critical modulator of inflammation and cell death in numerous tissues and diseases due to the cooperative activity of its deubiquitinating and ubiquitin ligase domains (4, 6). We recently showed that A20 attenuates the inflammatory response to *Porphyromonas gingivalis*, a keystone periodontal bacteria, by interfering with NF- κB signaling in macrophages and A20 deficiency promotes more severe periodontitis phenotype in a murine model of periodontitis (7). Gingival epithelial cells are among the first cell types encountered by bacteria and orchestrate a variety of responses by producing an array of inflammatory mediators and attract lymphocytes to the site of microbial insult. *P. gingivalis* can invade oral epithelial cells and induce production of inflammatory cytokines albeit much less quantities than other pathogens (8–11). Studies have been shown that while *P. gingivalis* initially employs strategies to subvert host immune responses for survival and a weak inducer of apoptosis, prolonged exposure of the oral mucosa to the bacterial and inflammatory insult further fuels inflammation and results in apoptosis and extensive tissue destruction, contributing to the chronicity of periodontitis (9, 12). A20 is implicated as a key regulator of epithelial cell biology in a variety of tissues (13–19). Clinically, A20 protein is expressed in the gingival epithelium, but there are still no studies investigating its function in the gingival epithelial cells (20). In this study, through elaborate use of gain and loss of function assays, we identified A20 as one of the novel regulators of inflammatory and apoptotic responses in gingival keratinocytes. Our results provide evidence of a previously unexplored immune regulatory pathway in gingival epithelial cell physiology and will have profound implications for the development of cell- and pathway specific immune therapies in the management of periodontitis.

## Materials and Methods

### Bacteria and Cell Culture

*Porphyromonas gingivalis* (strain ATCC33277) was maintained in Brain Heart Infusion supplemented with 0.05% Yeast Extract, 5 g/mL Hermin, 0.5 g/mL Vitamin K and 0.1% cysteine. The culture was placed in anaerobic chambers at 37°C. Bacteria were killed by heating at 80°C for 10 min (20). Heat- killed bacteria were used for all experiments except for *P. gingivalis* induced apoptosis. TIGKs (telomerase immortalized gingival keratinocytes) were kindly provided by Dr. Richard Lamont (University of Louisville). TIGKs have been established by immortalizing primary gingival epithelial cells and share similar morphology, karyotype, expression of cytokeratin and Toll-like receptors, and kinetics of invasion of *P. gingivalis* with parental cells and widely used in preclinical studies (21–23). TIGKs were maintained in LifeLine DermaLife medium with supplements (rh-insulin, L-glutamine, epinephrine, Apo-Transferrin, rh-TGF-α, Extract P, Hydrocortisone, LifeLine technology) as described previously (21).

### Enzyme Linked Immunosorbent Assay and Quantitative Real-Time (qRT) PCR

Culture supernatants were harvested after *P. gingivalis* infection (100 MOI) and analyzed using Human IL-6/IL-8 ELISA Ready-SET-Go!, 2nd Generation Kit (Invitrogen, Thermo Fisher Scientific) following users’ manual (7). Briefly, 96-well plates were coated with Capture antibody in coating buffer and incubate at 4°C overnight. After washing with wash buffer (0.05% Tween in PBS) and blocking with 1× Diluent, the supernatants were then added to the wells and incubated for 2 h in room temperature. Then Detection antibody was added and followed by Avidin incubation. The 96-well plates were washed with wash buffer as directed after each incubation. TMB buffer was added to the wells following stop solution addition. The plates were read at 450 nm with Multiskan MCC (Thermo Fisher Scientific). For qRT-PCR, RNAs in TIGKs were prepared with RNeasy Plus mini kit (Qiagen) using Qiacube (Qiagen) and processed following published protocols (7). cDNAs were synthesized from 800 ng of total RNA with High-Capacity cDNA Reverse Transcription Kit (Applied Biosystems) and were then used as templates for qRT-PCR with SYBR Green Master Mix (SABiosciences) on Applied Biosystems 7500. The primers used in qRT-PCR are listed in Table 1.

**TABLE 1:** Sequences for primers used in the study.

**qRT-PCR primers**	
hGAPDH-Forward	CAATGACCCCTTCATTGACC
hGAPDH-Reverse	TTGATTTTG GAGGGATCTCG
hA20-Forward	TTGTCCTCAGTTTCGGGAGAT
hA20-Reverse	ACTTCTCGACACCAGTTGAGTT
**sgRNA primers**	
sgRNA-s	CACCGAACCATGCACCGATACACAC
sgRNA-as	AAACGTGTGTATCGGTGCATGGTTC
**Screening primers**	
Cas9-screening-s	GAGGTGCTAAGATCTTTTGCCTACAGAC
Cas9-screening-as	GTAAAGGCCATTGGCAGAAC

### Western Blot

The cells were lysed with 1× RIPA buffer (diluted from 10× RIPA buffer, Cell Signaling Technology, #9806) or 1× SDS loading buffer and the supernatants were collected and applied to SDS-PAGE. Western blots were performed following the standard protocol as described (7, 20). Briefly, after SDS-PAGE, the proteins were transferred to PVDF membranes (Millipore). The membranes were blocked with 5% non-fat dry milk or BSA in TBST according to primary antibody preference. Followed by primary antibody incubation, the blots were washed with TBST buffer and incubated with secondary antibodies. The antibodies included: anti-TNFAIP3 (A20) antibody (D13H3, Cell Signaling Technology, 5630S, 1:1000), anti-β-Actin antibody (C4, Santa Cruz, sc-47778, 1:1000), anti-IκBα antibody (C21, Santa Cruz, SC-371, 1:1000), NF-κB p65 Antibody (F-6, Santa Cruz, SC-8008, 1:200), anti-p-NF-κB p65 antibody (27. Ser536, Santa Cruz, SC-136548, 1:500), anti-cleaved caspase 3 antibody (Cell signaling technology, # 9661S, 1: 500). Images were captured with G: BoxXX6 (Syngene) and band intensity was determined using ImageJ, version 1.4.3.67.

### A20 Knockdown in TIGKs

Telomerase immortalized gingival keratinocytes were transfected with scrambled siRNA (Qiagen, 1022076) or A20 siRNA (Santa Cruz, sc-37655) utilizing Lipofectamine RNAiMAX Transfection Reagent (Invitrogen) following users’ manuals. Briefly, the cells were seeded the day before transfection, and the medium were changed to Opti-MEM (Gibco) before transfection on the next day. 1.5 ul of 10 mM siRNA were diluted in Opti-MEM and combined with diluted Lipofectamine RNAiMAX and added to the cells after 5-min incubation. The medium was changed to growth medium 4 h after transfection. Knockdown was confirmed by Western blot. A20 knockdown with CRISPR/cas9 was performed as directed by Addgene lentivirus protocol. Single guide RNA (sgRNA) was designed following the guidelines provided by Zhang’s Lab and inserted into lentiCRISPR v2 vector. sgRNA sequences used were listed in Table 1. Synthetic DNA oligos were inserted to the digested LentiCRISPR v2 following the methods provided by Zhang’s lab (24). The construct of lentiCRISPR/cas9 A20-sgRNA (A20-sgRNA) was confirmed by sequencing and alignment by NCBI Basic Local Alignment Search Tool (BLAST). To confirm the mutation of *A20*, genomic DNA were purified and used as templates for PCR to amplify *A20* gene region with screening primers (sequences see Table 1). The PCR products were then inserted to pCR2.1 plasmid from TA Cloning Kit (Invitrogen, K2020-20) and transduced into competent *E. coli* cells. The plasmids were then purified and sequenced. The mutation is defined in 221A addition by alignment with NCBI BLAST.

### Lentivirus Preparation and Infection of TIGKs

Lentivirus packaging was performed as previously described (7). LentiCRISPR v2 was a gift from Feng Zhang (Addgene plasmid # 52961; http://n2t.net/addgene:52961; RRID: Addgene_52961). pRSV-Rev (Addgene plasmid # 12253; http://n2t.net/addgene:12253; RRID: Addgene_12253), pMDLg/pRRE (Addgene plasmid # 12251; http://n2t.net/addgene:12251; RRID: Addgene_12251), and pMD2. G (Addgene plasmid # 12259; http://n2t.net/addgene:12259; RRID: Addgene_12259) were gifts from Didier Trono (25). HEK293T were transfected with pRSV-Rev, pMDLg/pRRE, pMD2 and empty vector (lentiCRISPR/cas9 v2) or lentiCRISPR-sgRNA (for A20 knockdown), or pCDH-GFP or pCDH-A20 plasmids (for overexpression). Forty-eight hours post transfection, the culture medium was collected and incubated with TIGKs overnight. Twenty-four hours after infection, the cells were selected with Puromycin (1 ug/mL) for about a week and pooled together to be used for experiments. A20 knockdown with CRISPR/cas9 or A20 overexpression were confirmed with Western blots.

### Immunofluorescence

Telomerase immortalized gingival keratinocytes seeded in EMD Millipore^TM^ Millicell^TM^ EZ the day before experiment were infected with *P. gingivalis* the next day and harvested for analysis by immunofluorescence. Briefly, the cells on the slides were fixed in 4% paraformaldehyde for 10 min, followed by three times washing with PBS. The cells were then permeated with 0.02% Triton-X in PBS for 5 min. After wash with PBS, the cells were blocked with 3% BSA in PBS for 30 min and incubated with anti-NF-κB p65 antibody (Santa Cruz, sc-8008, 1: 250) for 1 h. After three times wash with PBS, the cells were incubated with Alexa Fluor 555 goat anti-mouse IgG and DAPI at room temperature in the dark for 45 min and washed three times with PBS. Fluorescent images were captured at 20× magnification with Axiovent 200M (Zeiss). Nuclear localization of NF-κB p65 was identified by colocalization of NF-κB p65 antibody and DAPI nuclear stain. Two fields were randomly selected, and more than 500 cells were counted and analyzed for each sample.

### Apoptosis Assay

A20-altered and A20-competent TIGKs were stimulated with live *P. gingivalis* (100 MOI) for 24 h. For TNF induced apoptosis, cells were treated with TNF (Peprotech, cat. #:300-01A, 50 ng/mL) for 24 h and cycloheximide (CHX) (Millipore, cat. #: C7698-1G, 3 ug/mL) for 4 h. All cells were collected, lysed and applied to SDS-PAGE. Protein levels of cleaved caspase 3 were assessed by Western blots. The levels of cytoplasmic histone-associated DNA fragments were determined with Cell Death Detection ELISA kit (Roche, cat. #: 11544675001) following users’ manual. As an alternative positive control for apoptosis, CPT induced apoptosis was performed. Cells were treated with 1 uM Camptothecin (CPT, Sigma, cat. #: C9911) for 6 h ours and the levels of cytoplasmic histone-associated DNA fragments were determined using ELISA.

### Statistical Analysis

Data were analyzed by one-way analyses of variance (ANOVA) using Tukey multiple comparisons or unpaired *t*-test with Mann-Whitney correction or Wilcoxon signed rank test using GraphPad Prism (version 8.0). Each experiment was repeated at least three times and a *p* value of ≤ 0.05 was considered significant.

## Results

### A20 Expression Increased in Gingival Keratinocytes Upon *Porphyromonas gingivalis* Infection

**FIGURE 1:**
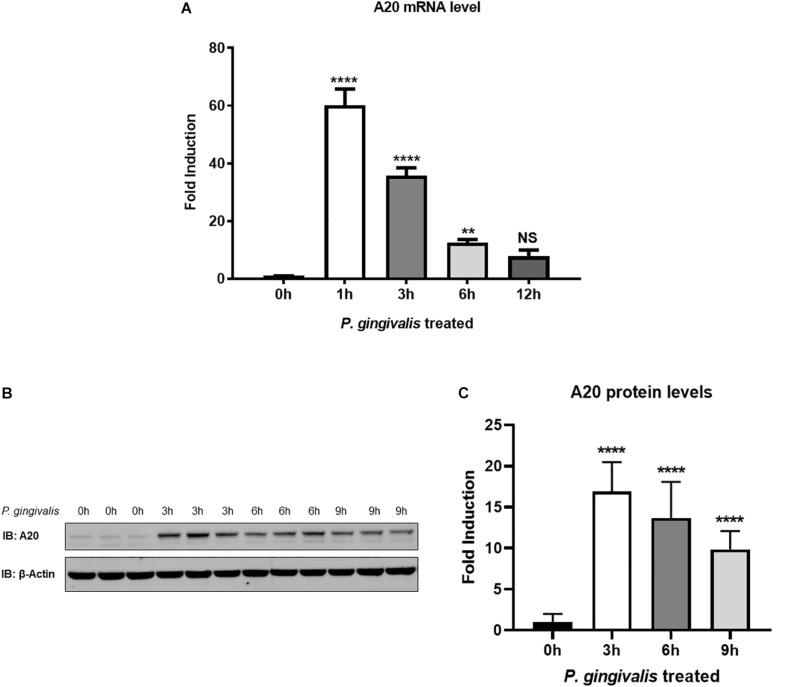
A20 expression is induced by *P. gingivalis* infection in human gingival keratinocytes. **(A)** Quantitative polymerase chain reaction gene expression analysis of A20 expression in TIGKs after *P. gingivalis* (100 MOI) infection for indicated time. Each experiment was performed three times independently and representative blots were shown. NS, not significant, **P ≤ 0.01, ****P ≤ 0.0001. Values are presented as mean ± SD. Representative blots **(B)** and corresponding quantification **(C)** show that the expression of A20 was induced by *P. gingivalis*. Values are presented as mean and standard error.

We first sought to examine the A20 expression in TIGKs following *P. gingivalis* infection. TIGKs are well established cells displaying morphology and protein expression profiles similar to the parental gingival epithelial cells, making them a perfect tool for preclinical assays (21, 26). An early up-regulation of A20 mRNA was observed in *P. gingivalis* infected cells, with expression peaking at 1 h post infection and gradually dropping yet remaining significantly elevated up to 6 h (Figure 1A). Similarly, the amount of A20 protein was elevated significantly upon *P. gingivalis* infection, demonstrating a peak increase at 3 h and subsequently showing a significant decrease at 6 and 9 h (Figures 1B,C). These findings revealed the pattern of A20 expression in gingival keratinocytes in response to oral bacterial challenge with notable increase at the early stages of infection.

### A20 Regulates Inflammatory Cytokine Production in Gingival Keratinocytes Following *Porphyromonas gingivalis* Infection

**FIGURE 2:**
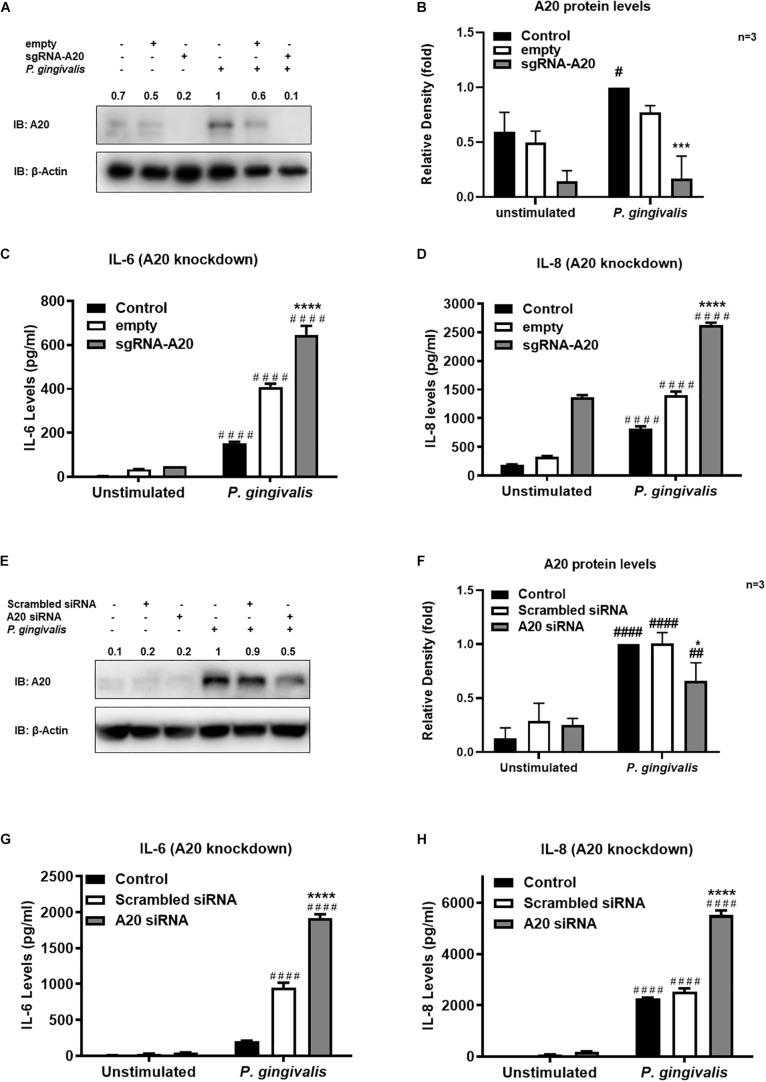
A20 depletion increases inflammatory cytokine production in human gingival keratinocytes. ELISA assays detecting cytokine production of TIGKs with depleted A20 expression or control cells infected with *P. gingivalis*. TIGKs with lentivirus containing sgRNA targeting A20 (sgRNA-A20) or empty vector (empty) were infected with *P. gingivalis* (100 MOI) for 6 h. Cells were lyzed for indicated protein levels, represent Western blots were shown **(A)**. Relative protein levels for three (independent experiments were plotted with mean ± SD **(B)**. IL-6 **(C)** and IL-8 **(D)** levels in the supernatants were determined with ELISA. TIGKs were transfected with siRNA targeting A20 or scrambled siRNA and infected with *P. gingivalis* (100 MOI) for 6 h. Cells were lyzed for indicated protein levels, represent Western blots were shown **(E)**. Relative protein levels for three independent experiments were plotted with mean ± SD **(F)**. The levels of IL-6 **(G)** and IL-8 **(H)** in the supernatants were determined with ELISA. Each experiment was performed three times independently. Bar graphs: Values are presented as mean and standard error. ^#^Unstimulated cells versus corresponding *P. gingivalis* infected cells (*P* ≤ 0.05); *P. gingivalis infected A20 depleted cells compared to infected controls (*P* ≤ 0.05); ^####^Unstimulated cells versus corresponding *P. gingivalis* infected cells (*P* ≤ 0.0001); ****P. gingivalis infected A20 depleted cells compared to infected controls (*P* ≤ 0.0001). ^##^Unstimulated cells versus corresponding *P. gingivalis* infected cells (*P* ≤ 0.01).)

**FIGURE 3:**
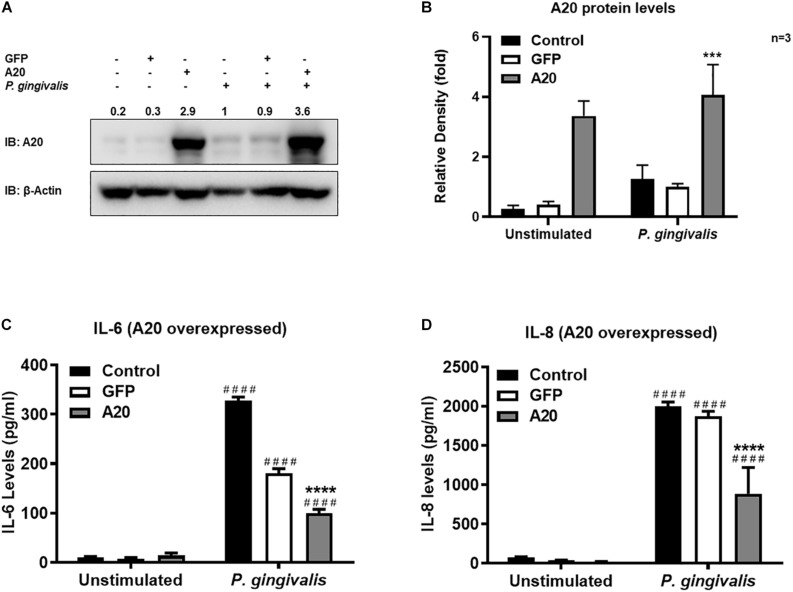
Exogenous expression of A20 inhibits inflammatory cytokine production in human gingival keratinocytes. ELISA cytokine production assays of TIGKs with exogenous A20 expression or control cells infected with *P. gingivalis*. TIGKs were infected with lentivirus expressing A20 or GFP and infected with killed *P. gingivalis* (100 MOI) for 6 h. Cells were lyzed for indicated protein levels, represent Western blots were shown **(A)**. Relative protein levels for three independent experiments were plotted with mean ± SD **(B)**. IL-6 **(C)** and IL-8 **(D)** levels in the supernatants were determined with ELISA. Each experiment was performed three times independently and representative experiments were shown. Bar graphs: Values are presented as mean and standard error. ^####^Unstimulated cells versus corresponding *P. gingivalis* infected cells (*P* ≤ 0.0001); ****P. gingivalis infected A20 depleted cells compared to infected controls (*P* ≤ 0.0001); ***P. gingivalis infected A20 depleted cells compared to infected controls (*P* ≤ 0.001).

Gingival keratinocytes form the first physical barrier between the oral microenvironment and the underlying tissues. Cytokines and chemokines such as IL-6 and IL-8 produced by the epithelial cells in response to resident bacteria recruit leukocytes to infected sites to engulf pathogens and infected cells. To determine the function of A20 in gingival keratinocytes, our next set of experiments employed loss and gain of function assays in which A20 knockdown (siRNA, or the CRISPR/Cas9 genome editing and lentivirus transduction) and overexpressing (lentivirus transduction) systems were constructed followed by oral bacterial infections. We first confirmed the knockdown efficiency and noted decreased A20 protein levels in TIGKs transduced with lentivirus containing sgRNA targeting A20 gene (Figures 2A,B) and in the cells transfected with short interfering RNA (siRNA) targeting A20 compared to the controls (Figures 2E,F). Consistent with the other cells, basal levels of A20 expression in gingival keratinocytes were also low (16, 19, 27, 28). *P. gingivalis* has been identified as a weak cytokine/chemokine inducer in gingival epithelial cells (7, 29, 30). Our results were also in agreement with the previous reports and revealed slight yet significant increase in IL-6 and IL-8 levels following bacterial infection in all the cells compared to unstimulated controls. Further analyses revealed significantly increased IL-6 and IL-8 production upon *P. gingivalis* infection in A20 depleted TIGKs compared to the control cells transduced with empty vector (Figures 2C,D) or transfected with scrambled siRNA (Figures 2G,H). Taken together, these results indicated that A20 depletion potentiates cytokine production in gingival keratinocytes. It is of note that empty vector transfected cells also produced increased IL-6 and IL-8 compared to A20 competent cells possibly due to the effect of cell manipulations (e.g., Lentivirus infection) on cell phenotype which were reported in previous studies. Still there was significantly more cytokine release in A20 depleted gingival keratinocytes compared to empty vector transfected controls (31). In fact, treatment with scrambled siRNA also resulted in higher cytokine production again consistent with previous investigations which reported various effects of exogenous DNA or RNA delivery on cellular phenotype (32–38). We next assessed the effect of A20 overexpression on inflammatory mediator production in TIGKs transduced with lentivirus expressing A20 or GFP. We established A20 expressing cells with increased A20 levels compared to GFP transduced or control cells (Figures 3A,B). Verifying the data obtained from the loss of function assays, TIGKs overexpressing A20 produced significantly less IL-6 and IL-8 following bacterial infection compared to the GFP expressing and A20 competent control cells (Figures 3C,D). While IL-6 production was surprisingly less in GFP expressing infected cells compared to A20 competent cells (Figure 3C), we did not observe similar trend for IL-8. Moreover, the levels of both IL-6 and IL-8 were significantly less following bacterial challenge in A20 overexpressing cells versus GFP expressing cells. Collectively, these results revealed A20 as one of the regulators of inflammation in gingival keratinocytes.

### A20 Regulates Inflammation by Interfering With NF-κB Signaling in Gingival Keratinocytes

**FIGURE 4:**
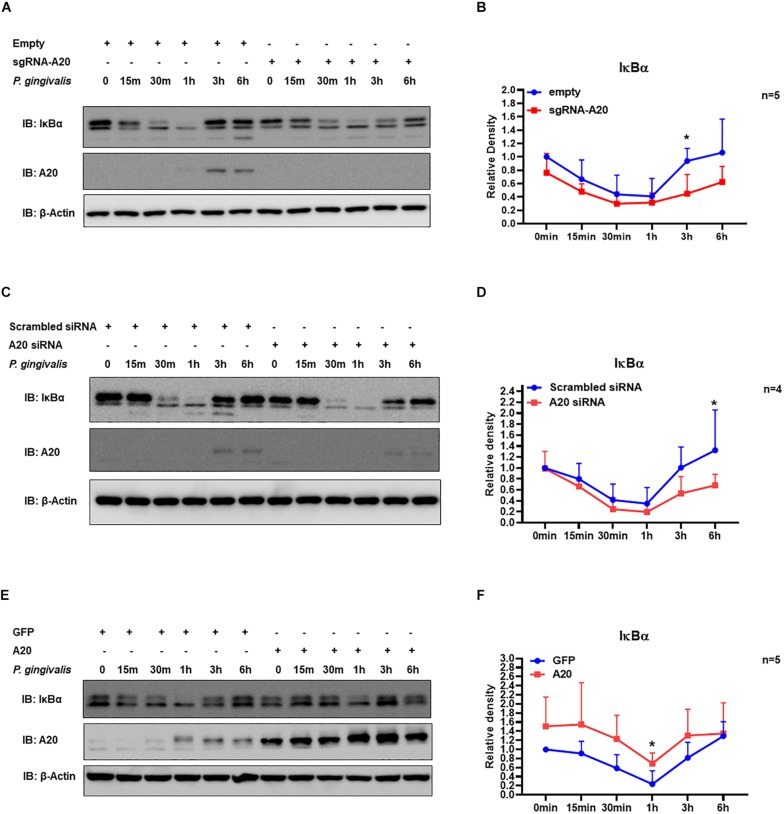
A20 regulates TIGKs inflammatory response to *P. gingivalis* infection through NF-kB signaling. Western blots detecting IκBα levels in TIGKs with altered A20 expression or control cells infected with *P. gingivalis*. Representative blots of IκBα levels in A20 depleted TIGKs infected with *P. gingivalis* **(A,C)** Relative density of IκBα levels from four or five independent experiments were plotted with mean and standard errors **(B,D)**. Representative blots of IκBα levels **(E)** in A20 overexpressing TIGKs infected with *P. gingivalis* and relative density of IκBα levels from five independent experiments as indicated were plotted with mean and standard errors **(F)**. Control or A20 expression altered TIGKs were infected with *P. gingivalis* (100 MOI) for the indicated time courses and the cells were harvested and lyzed and applied for protein assays with Western blots. Each experiment was performed independently, and representative blots were shown. Values are presented as mean and standard error. *P <0.05.

Our data indicated that A20 can suppress *P. gingivalis* induced cytokine production in gingival keratinocytes. NF-κB activation plays a key role in coordinating the expression of a variety of cytokines/chemokines in many chronic inflammatory diseases including periodontitis (1, 39). The primary mechanism for canonical NF-κB activation is the inducible degradation of IκBα. Following cellular stimulation by extracellular stimuli, IκBα is phosphorylated and then subsequently degraded in the cytoplasm. The eventual re-accumulation of IκBα drives the inhibition of NF- κB leading to decreased cytokine production. Hence, we first measured IκBα protein levels at different times for up to 6 h following *P. gingivalis* infection. The results showed rapid degradation of IκBα as early as 15 min followed by its recovery at later time points for all the cells tested (Figures 4A–F). This pattern was consistent with the established models of stimulus-induced degradation of IκBα followed by its re-accumulation and indicated NF-κB activation in response to *P. gingivalis* (40–43). To assess whether A20 regulates inflammatory response in gingival keratinocytes by modulating NF-κB activity, we monitored the degradation of IκBα in A20 depleted (A20 sgRNA and A20 siRNA) and A20 overexpressing (A20) TIGKs. Immunoblotting analyses of cell lysates isolated from *P. gingivalis* infected TIGKs revealed rapid IκBα degradation in A20 depleted cells within 15-min post-infection which was followed by its recovery to near resting levels by 6 h (Figures 4A–D). Representative blots are shown in Figure 4 accompanied by graphs plotting the values for all the experiments. Specifically, IκBα levels were significantly decreased for approximately 1.5–2 fold in A20 depleted cells compared to control cells after 1 h post infection (Figures 4B,D). In marked contrast, A20 overexpressing TIGKs displayed increased levels of IκBα after *P. gingivalis* infection compared to control cells (Figure 4F). These results indicated that A20 regulates NF-κB activity in gingival keratinocytes during bacterial exposure.

**FIGURE 5:**
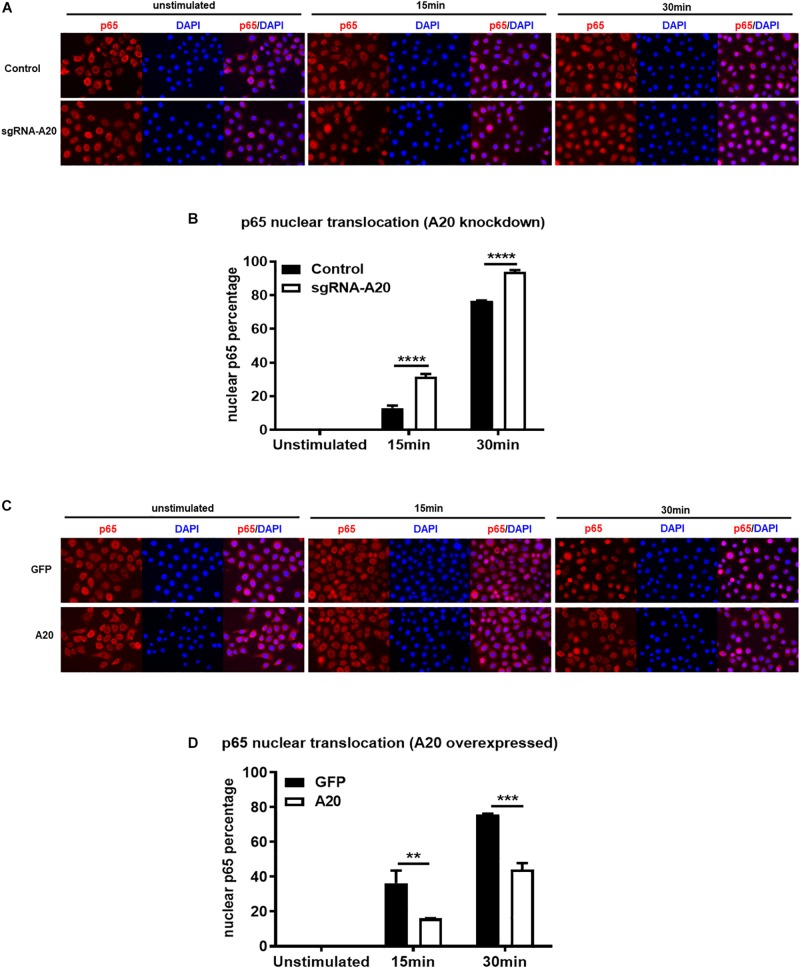
A20 diminishes p65 nuclear translocation in response to *P. gingivalis* infection in TIGKs. Immunofluorescence of p65 in TIGKs with altered A20 expression or control cells infected with *P. gingivalis*. p65 were stained with p65 antibody and then Alex-Fluor555 (red) and the nucleus were stained with DAPI (blue). Control or A20 expression altered TIGKs were infected with *P. gingivalis* (100 MOI) for indicated time courses and images were captured with Axiovent 200M. Representative images of control or A20 depleted TIGKs **(A)** and the plotted percentage of p65 in the nucleus **(B)**. Representative images of GFP control or A20 overexpressing TIGKs **(C)** and the plotted percentage of p65 in the nucleus **(D)**. Each experiment was performed three times independently and representative experiments were shown. Bar graphs: Values are presented as mean and standard error. **P ≤ 0.01, ***P ≤ 0.001, and ****P ≤ 0.001.

**FIGURE 6:**
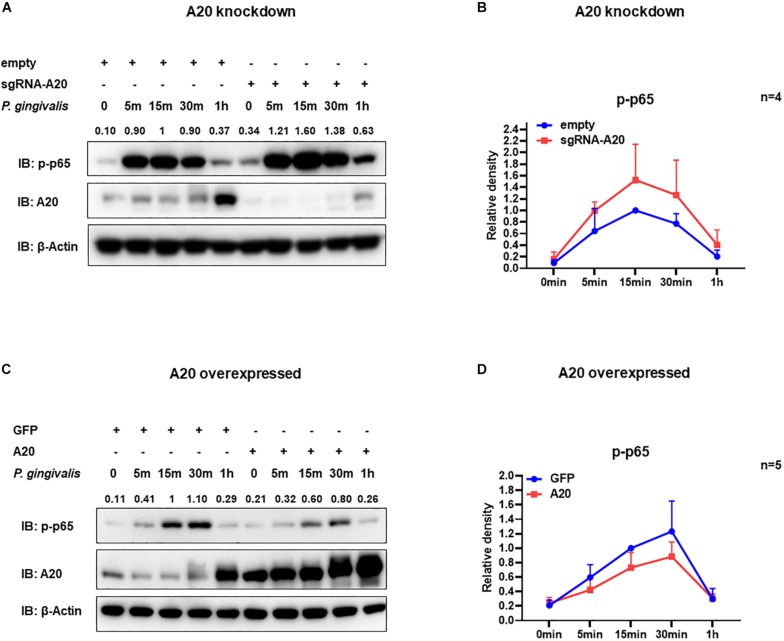
A20 diminishes p65 phosphorylation in TIGKs infected with *P. gingivalis*. Western blots detecting phosphorylated p65 (p-p65) in TIGKs with altered A20 expression or control cells with *P. gingivalis* infection. Representative blots of p-p65 in TIGKs or TIGKs with A20 knock-down **(A)** or A20 over-expression **(C)** with *P. gingivalis* (100 MOI) for indicated time points and the levels for indicated proteins were blotted. Relative density of p-p65 levels from four or five independent experiments were plotted with as mean and standard errors **(B,D)**.

Degradation of IκBα is a prerequisite for the NF-κB nuclear translocation. To further verify the role of A20 in modulating NF-κB activity in TIGKs, we also monitored NF-κB subunit p65 nuclear translocation using immunofluorescence microscopy. Consistent with the data obtained with immunoblotting, an increased percentage of nuclear p65 was observed in A20 depleted cells (Figures 5A,B), whereas exogenous expression of A20 resulted in a lower percentage of p65 nuclear translocation compared to the control cells following *P. gingivalis* infection (Figures 5C,D). In Addition, phosphorylated p65 (p-p65) were increased in A20 depleted cells compared to A20 competent cells at 15- and 30-min post-infection further substantiating the significance of A20 in this pathway (Figures 6A,B). While the observed differences in p-p65 levels were not significant compared to A20 competent cells, the results showed a similar trend in every experiment indicating that diminished A20 levels can have a profound effect on inflammatory responses in the oral cavity. Similar to the results obtained with IκBα, we also detected decreased levels of p-p65 in A20 overexpressing cells after 15 min post infection (Figures 6C,D). Taken together, our results revealed that A20 regulates oral bacteria-induced inflammatory cytokine axis in gingival keratinocytes via interfering with NF-κB signaling cascade.

### Lack of A20 Sensitizes Gingival Keratinocytes to Apoptosis Following *Porphyromonas gingivalis* Infection and TNF/CHX Exposure

**FIGURE 7:**
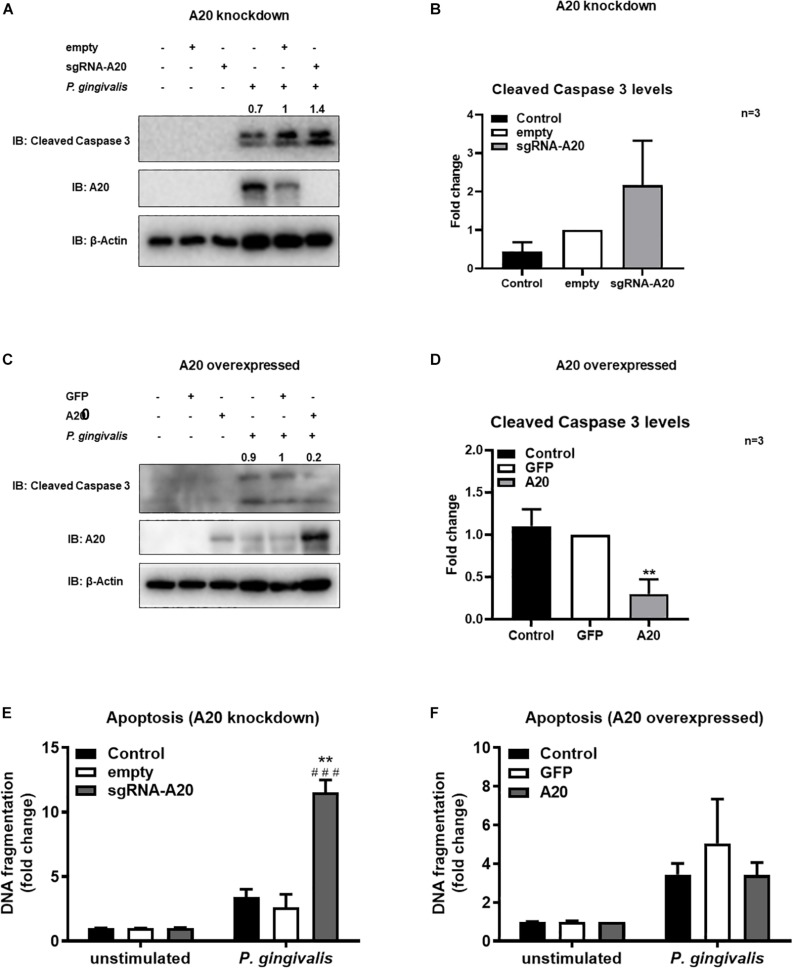
A20 restricts apoptosis in gingival keratinocytes following *P. gingivalis* infection. Western blots detecting cleaved caspase 3 levels in TIGKs with altered A20 expression or control cells with *P. gingivalis* infection. Representative blots of cleaved caspase 3 levels in TIGKs with control or A20 knock-down **(A)** and A20 over-expression **(C)** infected with *P. gingivalis* (100 MOI) for 24 h. Relative density of cleaved caspase 3 levels from three independent experiments were plotted with mean and standard errors **(B,D)**. **P. gingivalis infected A20 overexpressed cells compared to infected controls (*P* ≤ 0.01). ELISA detecting cytoplasmic histone associated DNA fragments in TIGKs. A20 depleted and control cells **(E)**, or A20 over-expressing cells and control cells **(F)** were infected with *P. gingivalis* for 24 h and applied to Cell Death Detection ELISA. Each experiment was performed three times and representative experiments were shown. ^###^Unstimulated cells versus corresponding *P. gingivalis* infected cells (*P* ≤ 0.001); **P. gingivalis infected A20 depleted cells compared to infected controls (*P* ≤ 0.01).

Besides promoting an inflammatory environment leading to tissue destruction, the oral mucosal milieu in chronic periodontitis can modulate apoptotic pathways as well (44, 45). Maintenance of the integrity of the gingival epithelium is therefore crucial to resist the external stress imposed to the oral mucosa by continuous microbial and inflammatory insult and masticatory forces. As A20 functions as a regulator of cell death in a variety of tissues, we next assessed its function in the regulation of apoptosis in TIGKs. Briefly, A20-competent and -altered TIGKs were challenged with live *P. gingivalis* (100 MOI) for 24 h and apoptosis was determined through monitoring cleavage of caspase 3 and detecting cytoplasmic histone associated DNA using ELISA (46–49). *P. gingivalis* is considered as a weak inducer of apoptosis and our results were consistent with this notion revealing minimum to no apoptosis in A20 competent TIGKs following bacterial infection (Figures 7A–F). On contrary, A20 depleted cells presented increased levels of cleaved caspase 3 and DNA fragmentation compared to control cells (Figures 7A,B,E). Consistently, the cells overexpressing A20 displayed decreased cleaved caspase 3 levels and DNA fragmentation upon *P. gingivalis* infection compared to GFP-transduced cells (Figures 7C,D,F). While GFP treatment affected the cell phenotype and the GFP transfected control cells exhibited more apoptosis, the A20 overexpressing cells showed similar phenotype as the wild type cells. These results collectively suggest that A20 serves as one of the regulators of apoptotic response in gingival keratinocytes and that lack of A20 sensitizes cells to apoptosis.

**FIGURE 8:**
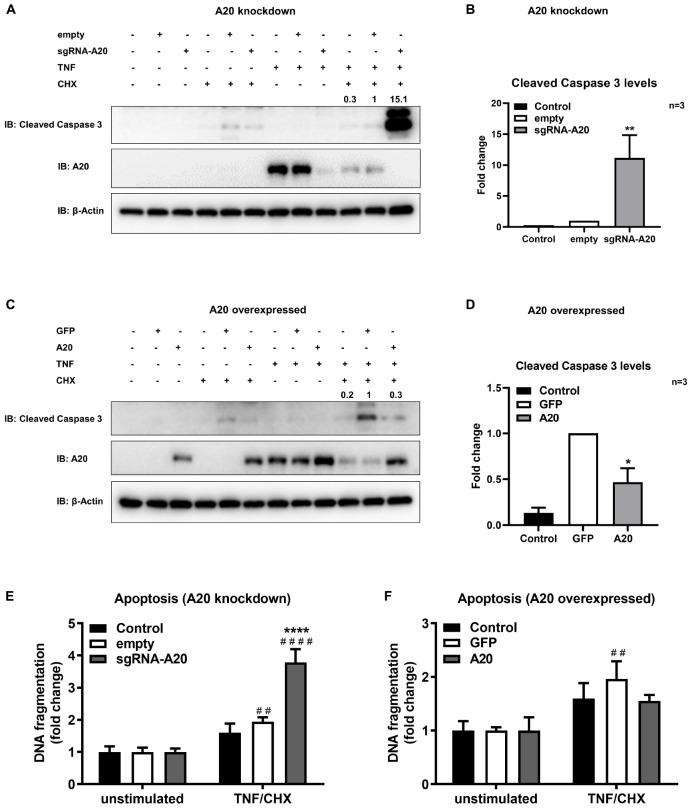
A20 restricts apoptosis in gingival keratinocytes induced by TNF/CHX treatment. Western blots detecting cleaved caspase 3 levels in TIGKs with altered A20 expression or control cells with TNF/CHX treatment. Representative blots of cleaved caspase 3 levels in TIGKs with control or A20 knock-down **(A)** and A20 over-expression **(C)** with TNF/CHX treatment (TNF 50 ng/mL 24 h, CHX 3 ug/mL 4 h). Relative density of cleaved caspase 3 levels from independent experiments were plotted with mean and standard errors **(B,D)**. **TNF/CHX treated A20 depleted cells compared to infected controls (*P* ≤ 0.01). *TNF/CHX treated A20 overexpressed cells compared to infected controls (*P* ≤ 0.05). ELISA detecting cytoplasmic histone associated DNA fragments in TIGKs. A20 depleted and control cells **(E)**, or A20 over-expressing cells and control cells **(F)** were treated with TNF/CHX (TNF 50 ng/mL for 24 h, CHX 3 ug/mL for 4 h) and applied to Cell Death Detection ELISA. Each experiment was performed three times independently and the representative experiments were shown. ^#^Unstimulated cells versus corresponding TNF/CHX treated cells (^##^*P* ≤ 0.01, ^####^*P* ≤ 0.0001); ****TNF/CHX treated A20 depleted cells compared to TNF/CHX treated controls (*P* ≤ 0.0001).

To further confirm the function of A20 in apoptosis we used a model mimicking the inflammatory microenvironment of the oral cavity and exposed TIGKs to the combination of TNF/CHX treatment which has been established as a potent inducer of apoptosis (48, 50). In previous reports, it has been shown that TNF alone is not sufficient to induce apoptosis in all cell lines and therefore combination of TNF and CHX has been commonly used to study apoptosis (51, 52). Our results showed minimum to no apoptotic response upon TNF/CHX treatment in A20 competent TIGKs as assessed by the levels of cleavage of caspase 3 and cytoplasmic histone associated DNA (Figures 8A–F) whereas there were about 10-fold increased levels of cleaved caspase 3 in A20-depleted cells compared to the control cells (Figures 8A,B). Similarly, A20 depleted cells exposed to TNF/CHX also exhibited significantly increased cytoplasmic histone associated DNA fragments compared to unstimulated cells and infected control cells supporting the role of A20 in cell fate response (Figure 8E). On the contrary, the cells overexpressing A20 presented with decreased cleaved caspase 3 levels and DNA fragmentation following TNF/CHX treatment compared to control cells transduced with GFP (Figures 8C,D,F). Similar to the experiments utilizing bacterial challenge, we noted slightly more apoptosis following TNF challenge in GFP expressing control cells which was likely due to the effect of cell manipulations. Yet, the levels of cleaved caspase 3 and DNA fragmentation in A20 overexpressing cells were comparable to the levels in A20 competent cells indicating that A20 overexpression helped cells to recover and restored the original phenotype. To further substantiate the role of A20 in apoptosis, we performed ELISA detecting DNA fragmentation upon exposure to Camptothecin (CPT), a commonly used positive inducer for apoptosis. Corroborating with the results from bacterial and TNF/CHX induced apoptosis, A20 depleted cells displayed significantly more apoptosis indicated by DNA fragmentation in response to CPT treatment (Supplementary Figure S1A). However, A20 over-expressing cells displayed a phenotype similar to controls upon exposure to CPT indicating that A20 overexpression restores cellular phenotype and desensitizes cells to apoptosis (Supplementary Figure S1B).

Collectively, our results demonstrated that adequate A20 levels are essential to desensitize gingival keratinocytes to apoptosis induced by external stress and reveal A20 as one of the novel regulators of cell fate in the oral mucosa.

## Discussion

**FIGURE 9:**
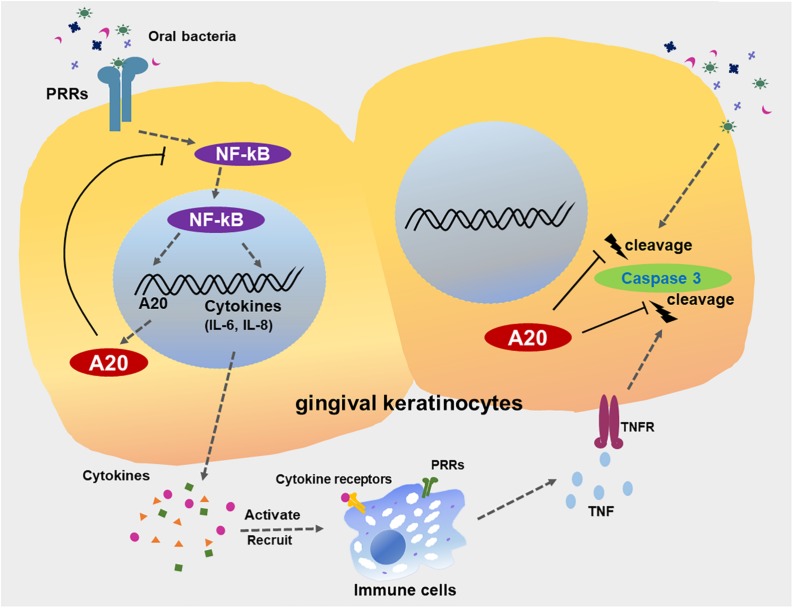
A20 restricts inflammatory responses and apoptosis in gingival keratinocytes. The engagement of microbial ligands with pattern recognition receptors (PRRs) on the surfaces of gingival keratinocytes initiates the activation of the NF-κB signaling cascade, promoting the production of downstream pro-inflammatory cytokines and chemokines, such as IL-6 and IL-8. Activation of NF-κB also induces the dramatic increase in A20 expression, resulting in a negative feedback loop responsible for the de-activation of NF-κB signaling and a return to oral mucosal tissue homeostasis. However, a failure to resolve inflammation results in the aberrant production of inflammatory mediators, which induce the rapid migration of more immune cells to the site of infection. These inflammatory processes could also drive neighboring cells to apoptosis, mediated primarily by further bacterial invasion and activation of several receptors including TNFR, thus contributing to the development of chronic inflammation seen in periodontitis. A20 subsequently restricts caspase 3 cleavage, thereby desensitizes gingival keratinocytes from apoptosis. Collective function of A20 facilitates the preservation of oral tissue homeostasis and maintenance of epithelial integrity.

Despite recent advances in the treatment of the periodontitis, management of the inflammatory component remains a major challenge for those experiencing severe and persistent forms of the disease. Targeting endogenous regulators as therapeutics is emerging as a promising strategy for numerous chronic diseases including periodontitis (53). We have previously reported that A20 attenuates the inflammatory response in the oral mucosa with a focus on A20 driven cellular function in macrophages (7). Besides its role in myeloid cells, A20 has also been shown to serve as a key regulatory molecule of epithelial cell physiology (13, 15, 16). Current study characterizes the function of A20 in gingival epithelial cell response to external stress and introduces a number of novel concepts to our understanding of the oral cavity microenvironment and the biological pathways which facilitate the maintenance of oral mucosal tissue homeostasis (Figure 9). First, this is the first report which reveals a specific role of an ubiquitin editing molecule in gingival keratinocyte inflammatory response and draws attention to the function of ubiquitination driven biological events in the milieu of periodontal inflammation. Second, our data indicates that sufficient A20 levels are essential in controlling apoptosis in response to microbial and inflammatory insult and emphasizes the significance of host specific factors in governing the epithelial cell fate in the oral mucosa. Third, our findings show that the pattern of A20 expression in response to oral bacterial challenge displays variation among different cells highlighting the importance of studying the cell-type specific A20 responses and their effect on disease phenotype to develop more targeted therapies. Fourth, we consistently noted less sensitivity to apoptosis in A20 competent gingival keratinocytes to all the external challenge which is likely related to their unique location in the body where they have to withstand constant exposure to external and internal stress from a wide plethora of sources affecting their phenotype.

These findings are consistent with the previous reports which revealed A20 as a major determinant of the disease characteristics and progression in several other immune and inflammatory conditions (5, 14, 16, 54–57). In the lung, airway epithelial cells of patients with asthma and cystic fibrosis were reported to express significantly less basal A20 levels than control airway epithelial cells and aberrant NF-κB activation in these patients was implicated with the A20 deficiency (58). It has been also shown that psoriatic skin lesions display decreased levels of A20 compared to healthy areas and A20 deficiency is associated with elevated inflammatory response in epidermal keratinocytes and increased susceptibility to severe experimental atopic dermatitis (56, 59). Similar characteristics were noted in the oral mucosa as well, where in spite of elevated A20 mRNA expression observed in periodontitis lesions, A20 protein levels remained comparable to healthy tissues (20). These observations suggested that A20 protein may not be sustained in ample quantity to facilitate the resolution of inflammation in periodontal tissues. Furthermore, it has been also shown that even a partial loss of A20 results with more severe periodontal disease phenotype in a murine model of periodontitis indicating that fluctuations in A20 levels can modulate inflammatory response in the oral cavity (7). The underlying mechanisms for A20 deficiency in different tissues including oral mucosa are yet to be identified but likely comprised of transcriptional and posttranscriptional regulation by complex factors involving genetic, epigenetic, systemic and/or environmental alterations (60–64). Here, we showed that lack of A20 in gingival keratinocytes significantly increased NF-κB activity and inflammatory cytokine and chemokine production in response to bacterial challenge. While similar findings were reported in macrophages and periodontal ligament cells, interestingly the pattern of A20 expression following infection varied between cell types (7, 65). Specifically, gingival keratinocytes responded to *P. gingivalis* infection with a rapid increase in A20 gene and protein levels which then followed by a significant drop to closer to basal levels whereas A20 levels were elevated and remained significantly higher in macrophages (7, 65). As periodontal tissues are formed by the cells of both myeloid and non-myeloid origin, future studies utilizing more targeted approaches, such as cell-lineage specific conditional knockout mice, are warranted to assess how differences in cell/tissue specific expression of A20 shape oral mucosal tissue homeostasis and influence periodontal disease phenotype. Collectively, our findings substantiate the role of A20 in the oral mucosa and emphasize that impaired A20 levels could have profound effect in different cellular responses and promote an unrestrained inflammatory response.

In addition to its key role in regulating inflammation, several lines of evidence implicate A20 in cell death response and report that A20 protects variety of cells from apoptosis (15–17, 66–69). For example, A20 ablation in both intestinal epithelial cells and myeloid cells in mice has been shown to induce spontaneous intestinal pathology due to hyper-inflammatory A20-deficient myeloid cells causing epithelial apoptosis and barrier destabilization (13). This self-perpetuating condition due to A20 deficiency causes severe inflammation and bacterial colonization in the gastrointestinal tract and somehow mimics some of the pathology seen in periodontitis. Thus, we pursued to investigate the role of A20 in apoptotic response in TIGKs. Our first set of experiments utilizing *P. gingivalis* as a model organism showed that while A20 competent cells remained resistant to bacteria-induced apoptotic response, A20 depletion triggered apoptosis upon exposure to bacteria. It is noteworthy that the impact of *P. gingivalis* on cell death has been controversial where studies reporting either apoptotic or anti-apoptotic effects. These differences have been attributed to the response being cell type and host resistance specific and/or associated with the length and amount of bacterial exposure and differences in the virulence of different bacterial strains (12, 44, 45, 49, 70–72). Here we consistently demonstrated that A20 competent cells exhibit minimal to no apoptosis upon *P. gingivalis* exposure consistent with previous reports. Yet, we were also unequivocally showed that insufficient A20 levels in gingival keratinocytes render the cells sensitive to bacteria-induced apoptosis and provided further evidence that host specific factors play important role in determining the cell fate against microbial insult. Our results combined with the previous studies highlight the significance of host-derived factors in controlling various cellular responses to resident microbiome and warrant future studies including other bacteria and strains to assess whether apoptotic response can be modulated through targeting A20.

Besides constant bacterial exposure, the inflammatory milieu of periodontal disease also provides a continuous cytokine insult to the oral mucosa. An additional established major apoptotic pathway operating through activation of caspases is the death receptor mediated, TNF or extrinsic pathway. In fact, *P. gingivalis*-induced fibroblast apoptosis is mediated to a large extent by TNF (48). Therefore, our following experiments included exposure of TIGKs to the combined treatment of TNF and CHX to induce a pro-apoptotic response using the amounts which were reported in previous reports. Similar to the results obtained with bacteria, TIGKs expressing sufficient A20 levels displayed only minimal apoptosis following TNF/CHX challenge whereas A20 depleted TIGKs showed a marked apoptotic response. It is important to note that GFP transfected cells showed higher apoptotic signals compared to A20 competent wild type cells whereas A20 overexpression reversed the effect and restored the original phenotype. Similar results were reported in intestinal epithelial cells and hepatocytes where A20 restricted cell death by inhibiting TNF-induced caspase 8 and RIPK1 kinase activity and caspase 3 activation, respectively (15, 73). Collectively, our results verify that A20 interferes with apoptotic response in TIGKs and uncover a previously unexplored pathway in gingival epithelial cell pathophysiology.

In summary, our results reveal A20 as a regulator of inflammation and cell fate in gingival keratinocytes (Figure 9). Gingival keratinocytes function as a critical interface between internal host tissues and the external environment. Loss of epithelial integrity from the induction of apoptosis facilitates further bacterial invasion. Compounding this diminished host response, prolonged apoptotic cell accumulation in inflamed sites of chronic periodontitis also results in increased inflammatory cell recruitment and further fuels inflammation, contributing to disease severity. In this study we used *P. gingivalis* as a model organism to assess the function of A20 in gingival keratinocytes as a proof of concept. Yet the resident oral microbiome is more complex and there are also reports showing modulation of A20 expression using probiotics (74, 75). Hence, future studies are warranted to determine the extent of A20 induction in response to other bacteria and whether A20 levels/activity can be altered through targeting microbiome. As we move to an era of personalized medicine, it will also be important to investigate the individual host and microbial factors as well as environmental exposures which can alter A20 levels/activity in the oral mucosa. A20 has been increasingly implicated as a therapeutic target in numerous conditions, some of which are associated with poor oral health (5). Therefore, it is likely that A20-targeted therapies can help to maintain the integrity of the gingival epithelium and promote the resolution of periodontal inflammation in the oral cavity thereby improving adverse clinical outcomes both at local tissues as well as distant sites.

## Data Availability Statement

The datasets analyzed in this article are not publicly available. Requests to access the datasets should be directed to corresponding author.

## Author Contributions

SS, YL, and EM designed the experiments, wrote the manuscript, and analyzed the data. YL, EM, NG, and X-JX performed the experiments.

## Conflict of Interest

The authors declare that the research was conducted in the absence of any commercial or financial relationships that could be construed as a potential conflict of interest.

## Supplementary Material

FIGURE S1 A20 restricts apoptosis in gingival keratinocytes induced by CPT treatment. ELISA detecting cytoplasmic histone associated DNA fragments in TIGKs with altered A20 expression or control cells with CPT treatment. A20 depleted and control cells **(A)**, or A20 over-expressing cells and control cells **(B)** were treated with 1 uM CPT for 6 h and applied to Cell Death Detection ELISA. Each experiment was performed three times independently and the representative experiments were plotted with mean and standard errors. ^#^Unstimulated cells versus corresponding CPT treated cells (^##^*P* ≤ 0.01, ^###^*P* ≤ 0.001, and ^####^*P* ≤ 0.0001); ^∗∗∗^CPT treated A20 depleted cells compared to CPT treated controls (*P* ≤ 0.001).
