# Supplementary figures and images for: A20 Restricts Inflammatory Response and Desensitizes Gingival Keratinocytes to Apoptosis
Source: Front Immunol. 2020 Mar 10;11:365. doi: 10.3389/fimmu.2020.00365 (PMC7078700; doi:10.3389/fimmu.2020.00365)

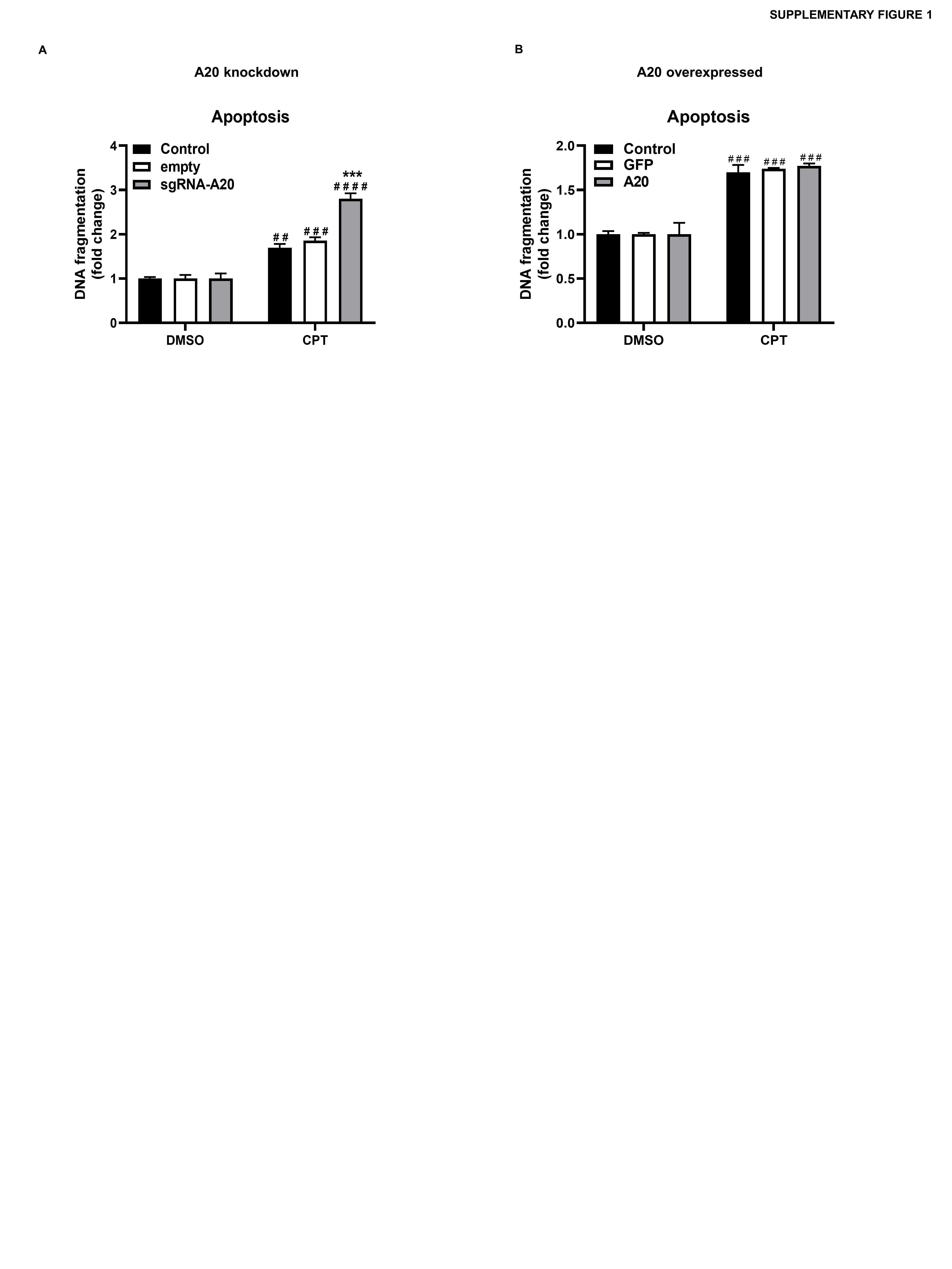

Supplement: FIGURE S1 — A20 restricts apoptosis in gingival keratinocytes induced by CPT treatment. ELISA detecting cytoplasmic histone associated DNA fragments in TIGKs with altered A20 expression or control cells with CPT treatment. A20 depleted and control cells (A), or A20 over-expressing cells and control cells (B) were treated with 1 uM CPT for 6 h and applied to Cell Death Detection ELISA. Each experiment was performed three times independently and the representative experiments were plotted with mean and standard errors. #Unstimulated cells versus corresponding CPT treated cells (##P ≤ 0.01, ###P ≤ 0.001, and ####P ≤ 0.0001); ∗∗∗CPT treated A20 depleted cells compared to CPT treated controls (P ≤ 0.001). [file Image_1.TIF]
